# A microprocessor controlled liquid chromatograph/atomic absorption sampling system

**DOI:** 10.1155/S1463924679000560

**Published:** 1979

**Authors:** Thomas M. Vickrey, William Eue.

**Affiliations:** Department of Chemistry Texas A & M University College Station Texas 77843 USA


on a centrifugal analyzer, since the amount of splashover depends upon the reagent as well as the suction force. No appreciable splashover should be tolerated because if the blank, standard or reference cuvettes are contaminated, all the results will be wrong.

ACKNOWLEDGEMENTS
The author is grateful to Mr. W. A. White for technical assistance, to V. A. Howe & Co. Ltd., for the loan of the instrument and to the Department of Health and Social Security for their permission to publish this article.

REFERENCES
[1] Anderson, N.G., (1969) *Analytical Biochemistry*, **28**, 545.
[2] Anderson, N.G., (1969) *Analytical Biochemistry*, **32**, 59.
[3] Anderson, N.G., (1969) *Science*, **166**, 317.
[4] Anderson, N.G., (1970) *American Journal of Clinical Pathology* **52**, 778.
[5] Henry, P. and Saunders, R.A., (1976) *Annals of Clinical Biochemistry*, **13**, 384.
[6] Young, D.S. and Gochman, N., in "Standard Methods of Clinical Chemistry" volume 7 Ed. Cooper G.R. 1972 Academic Press, p. 303.
[7] Broughton, P.M.G., Buttolph, M.A., Gowenlock, A.H., Neill, D.W. and Skentelbery, R.G., (1969) *Journal of Clinical Pathology* **22**, 278.

### A microprocessor controlled liquid chromatograph/atomic absorption sampling system

**Thomas M. Vickrey and William Eue.**

*Department of Chemistry, Texas A & M University, College Station, Texas 77843, USA.*

### Introduction

The need for molecular characterization of metal-containing species at the trace level has led to the interfacing of high resolution chromatographic techniques with element-specific detectors [1, 2, 3]. One such system is the interfacing of liquid chromatography (LC) to atomic absorption spectroscopy (AA). The resultant instrumentation (LCAA) is capable of specific characterization (speciation) of the metal-containing compounds and detection of the separate species at the nanogram level [3]. The application of LCAA with a graphite furnace atomic absorption spectrometer has been reported [1, 2]. The use of a graphite furnace atomic absorption spectrometer as a detector for a liquid chromatograph has technical problems associated with it. However, the ability to monitor biological transport of metal-containing species in the environment, speciate clinically important metal compounds, or to use metal complexation as a tag for nonmetal species which are otherwise difficult to detect [4], make this technique attractive.

This work presents a versatile controller for the sampling interface, using a 6800 based microprocessor system. This interface controller is software programable to operate either in the 'pulsed' mode, or in the 'total consumption' mode. The hardware and software descriptions are presented, as well as the results of sampling precision studies.

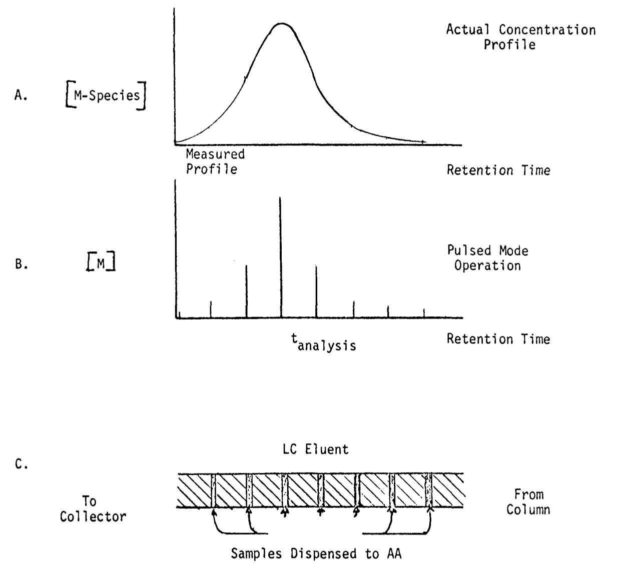

*Figure 1. Description of the "pulsed" sampling mode. A, actual concentration profile of metallospecies leaving the column. B, the measured concentration profile from the intermittent removal of aliquots of the eluent stream. C, a diagram of the eluent stream showing the aliquots which are removed and analyzed by the graphite furnace atomic absorption spectrometer.*

### Three sampling modes

There are three basic modes for LCAA operation: survey, pulsed and total consumption. In the survey mode the sample is chromatographed, fractions collected and the collected fractions analyzed for the metal of interest. This is the simplest form of the LCAA experiment and requires only the 'human' interface. The pulsed mode operates during the chromatographic run; the eluent stream is periodically sampled and the sample dispensed into a graphite furnace for the AA analysis cycle as shown in Figure 1. The term 'pulsed'

is used for this mode because the element concentration data can be obtained only once every 30-120 seconds depending on the element of interest.

This causes the chromatogram generated by the AA detector to have the appearance of 'pulsed' concentration varying with retention time or volume. The data obtained have the form of Figure 1B. An example of a pulsed mode chromatogram is shown in Figure 2. The most direct application of the pulsed mode of LCAA operation is to determine the presence and concentration of metal-containing species with unknown retention times, or reaction mixtures where the number of products and their identity is unknown (but all products are known to contain the metal of interest). The drawbacks of pulsed mode operation are that the mobile phase flow rates that can be used are very low. This is expecially true in the case of elements which have long AA analysis times (including the cooling of the graphite furnace). The flow rate must be low enough so that the product of one analysis time, $t_{analysis}$, (Figure 1B) and the flow rate does not exceed the peak width. Another related drawback is the small number of data points obtained to describe each peak's concentration profile (Figure 1B). In general, the number of data points per peak is between 4 and 8; also the chromatographic peak shape given by these peaks is not necessarily indicative of the shape of the concentration profile. Therefore, the third sampling method is designed to overcome these shortcomings.

The total consumption mode analyzes the total chromatographic peak by storage of the peak-containing eluent stream and subsequent dispensing of aliquots of the peak-containing eluent into the graphite furnace. In this mode the chromatographic peak is totally consumed. The temporary storage of

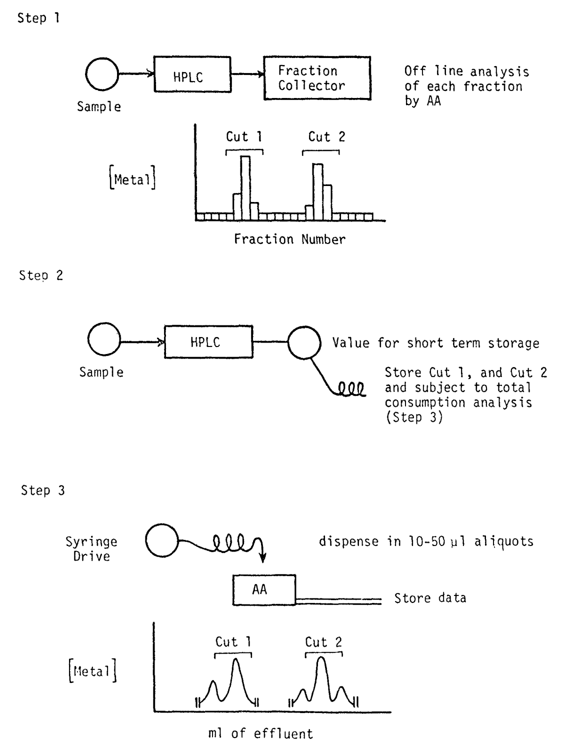

*Figure 3. Scheme for initial screening of samples and subsequent total consumption analysis.*

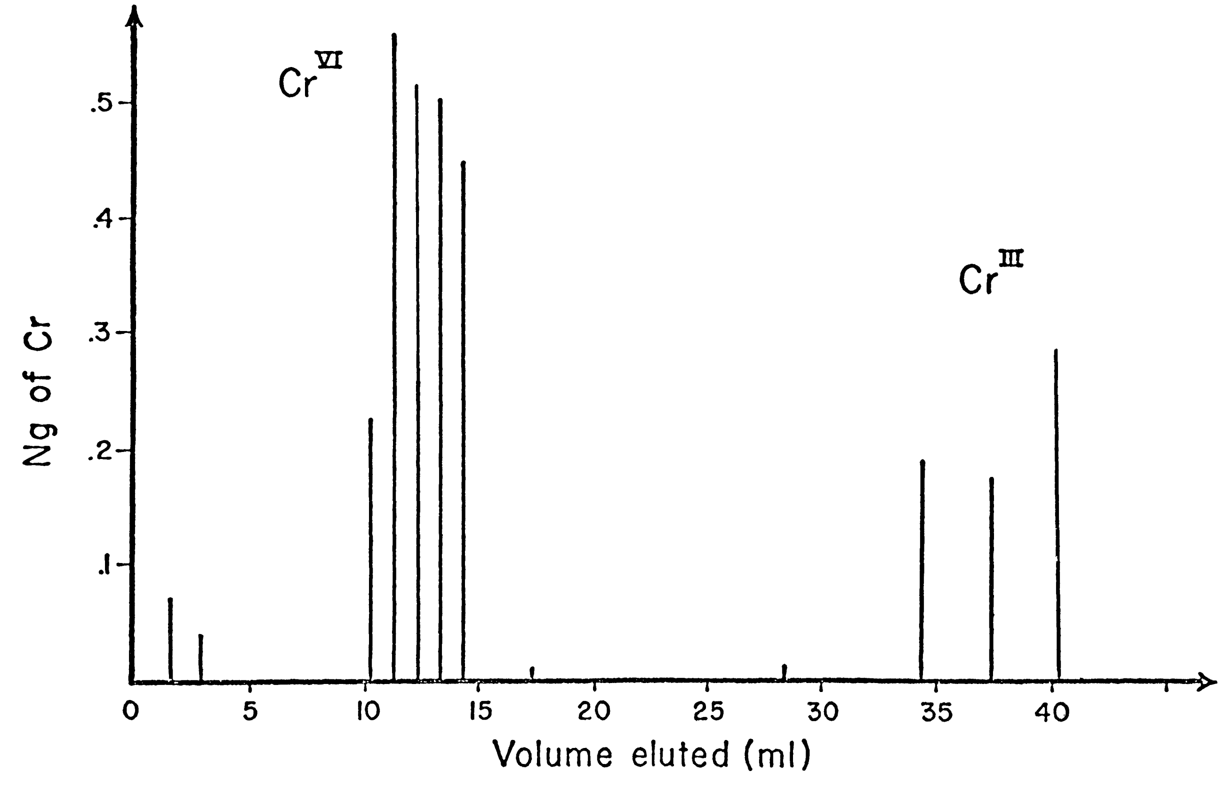

*Figure 2. LCAA output for the separation of a 1:1 mixture of Cr(III) and Cr(VI) on a cation exchange column (Partisil SCX) eluting with 0.1F acetate buffer, pH=4.3. Total chromium is equivalent to 40 ng.*

the peak-containing eluent in a capillary tube allows the chromatographic information to be retained and the analysis to be performed off-line. A flow scheme for the method is shown in Figure 3. This total consumption of the chromatographic peak by the graphite furnace analyzer lowers the limit of detection, and greatly increases the number of AA data points per chomatographic peak. Since the analysis is performed off-line the restrictions on the mobile phase flow rate are removed. Data from an experiment using this sampling method are shown in Figure 4. The drawbacks to this sampling method are that the retention time of the peak musι be known (at least approximately). Also the extra

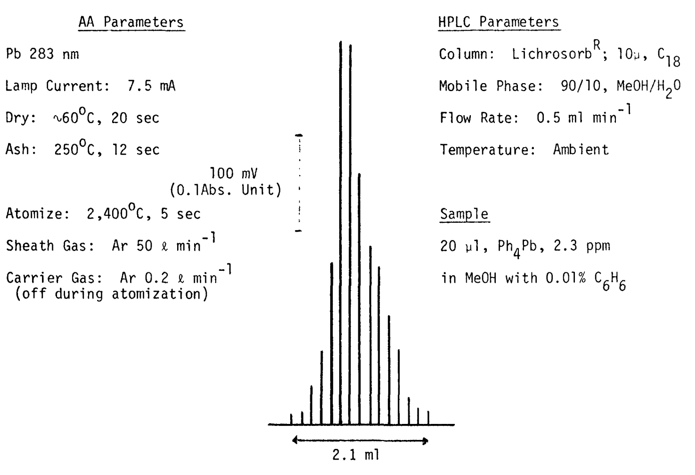

*Figure 4. Total consumption mode analysis of a tetraphenyl lead sample. LC and AA conditions as indicated. The capillary storage tube internal volume was 2.4 ml.*

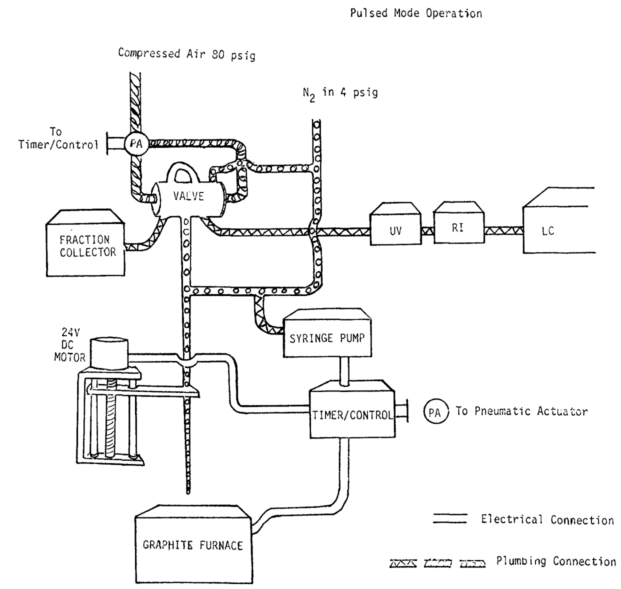

*Figure 5. Schematic diagram of liquid chromatograph-graphite furnace atomic absorption sampling system operating in the "pulsed mode". For total consumption mode operation, the sample storage loop is connected to the syringe pump and to the valve.*

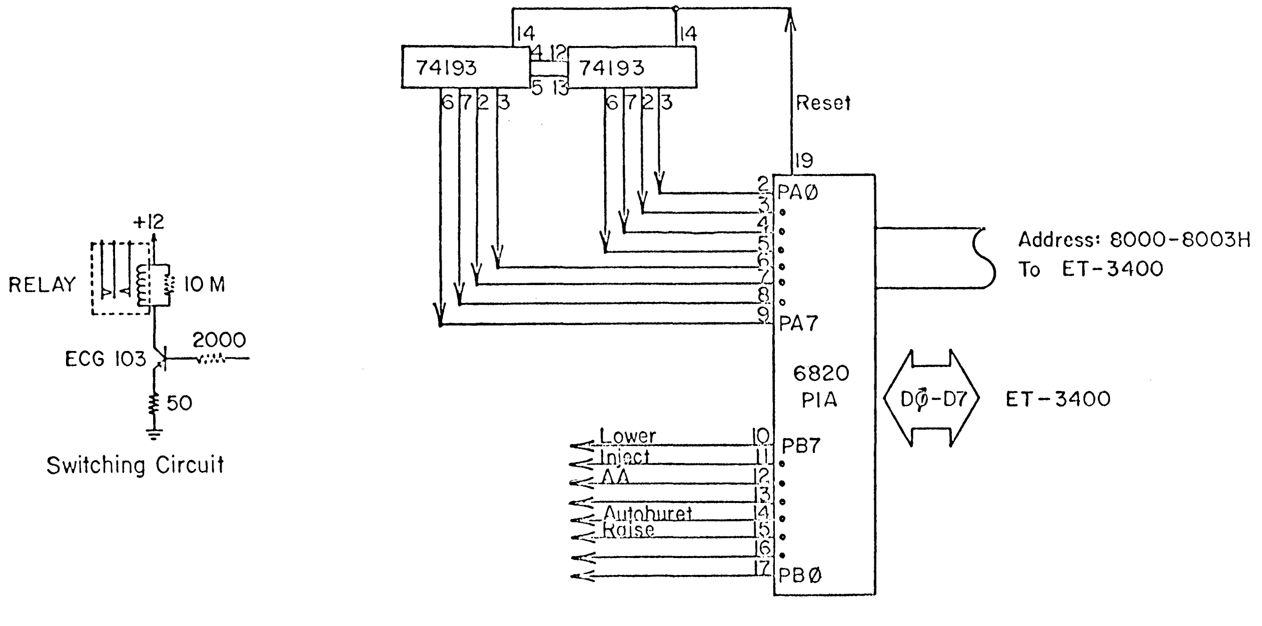

*Figure 6. Control unit for the sampling system. The timer/counters are read at the top of the PIA, the control bit are put out at the bottom. For details of each function see the text.*

column broadening effects due to the large 'dead volume' of the storage tube are not negligible, but the peak width increase can be corrected in the data analysis.

The last two sampling modes described above are complementary. The pulsed mode is most useful for exploratory studies and the total consumption mode is more suited to routine analysis of samples when the identity of the components is known. The interface system used should be capable of operation in any of the modes described above.

### Instrumentation and procedures

To this end, the microprocessor control LCAA interface has been developed and is shown diagramatically in Figures 5 and 6. The controller consists of (1) the Motorola 6800 based Heathkit microprocessor trainer, (2) external clock (3) electronic interface to the dispenser and to the AA, and (4) the dispenser system [1]. The microprocessor and peripheral interface adapter generate an 8 bit control pattern. This control pattern is maintained for the time programmed

**Table 1. Microprocessor control program listing**

```

                                       NAM          AACONTR

            **INITIALIZATION OF PIA

0000   CE 0004   START                 LDX          #$0004

0003   FF 8000                         STX           $8000

0006   CE FF04                         LDX          #$FF04

0009   FF 8002                         STX           $8002

            **CONTROL ROUTINE LOWER RACK

000C   86 01                           LDA A        #$0001      BIT PATTERN

000E   B7 8002                         STA A         $8002      LOWER RACK

0011   C6 05                           LDA B        #$05        TIME=5 SEC.

0013   BD 0120                         JSR INIT                 INITIALIZE CLOCK

0016   BD 0100                         JSR TIMEER               TIME SEQ.

            **CONTROL ROUTINE INJECT SAMPLE

0019   86 02                           LDA A        #$02        BIT PATTERN

001B   B7 8002                         STA A         $8002      INJECT

001E   C6 OA                           LDA B        #$OA        TIME=10 SEC.

0020   BD 0100                         JSR TIMER                TIME SEQUENCE

            **CONTROL ROUTINE FOR FIRST DRY CYCLE

0023   86 04                           LDA A        #$04        BIT PATTERN

0025   B7 8002                         STA A         $8002      AA ON

0028   C6 01                           LDA B        #$01        TIME=1 SEC.

002A   BD 0100                         JSR TIMER                TIME SEQUENCE

            **CONTROL SEQUENCE FOR DRYING TIME

002D   86 CO                           LDA A        #$CO        BIT PATTERN

002F   B7 8002                         STA           $8002      TIME DRY

0032   C6 0A                           LDA B        #$0A        TIME=10 SEC.

0034   BD 0100                         JSR TIMER                TIME SEQUENCE

            **CONTROL SEQUENCE FOR ENDING DRY CYCLE

0037   86 04                           LDA A        #$04        BIT PATTERN

0039   B7 8002                         LDA A        #$04        BIT PATTERN

003C   C6 01                           LDA B        #$01        TIME=1 SEC.

003E   BD 0100                         JSR TIMER                TIME SEQUENCE

            ** CONTROL SEQUENCE FOR SECOND INJECTION

0041   86 10                           LDA A        #$10        BIT PATTERN

0043   B7 8002                         STA A         $8002      INJECT

0046   C6 02                           LDA B        #$02        TIME= 2 SEC.

0048   BD 0100                         JSR TIMER                TIME SEQUENCE

            **CONTROL SEQUENCE TO RAISE RACK AND RESTART AA

004B   86 24                           LDA A        #$24        BIT PATTERN

004D   B7 8002                         STA A         $8002      RAISE & START

0050   C6 03                           LDA B        #$03        TIME=3 SEC.

0052   BD 0100                         JSR TIMER                TIME SEQUENCE

            **CONTROL SEQUENCE TO TIME AA CYCLE

0055   86 CO                           LDA A        #$CO        BIT PATTERN

0057   B7 8002                         STA A         $8002      TIME CYCLE

005A   C6 64                           LDA B        #$64        TIME=100 SEC.

005C   BD 0100                         JSR TIMER                TIME SEQUENCE

005F   7E 0000                         JMP START                RETURN

            **TIME SUBROUTINE

0100   B6 8000   TIMER                 LDA A         $8000      GET TIME

0103   01                              NOP

0104   11                              CMP                      COMPARE B WITH A

0105   26 F9                           BNE TIMER                BRANCH BACK

0107   86 00                           LDA A        #$00        ZERO BUFFER

0109   B7 8002                         STA A         $8002      OUTPUT

010C   BD 0120                         JSR           RESET      RESET CLOCK

010F   39                              RTN                      RETURN

            **CLOCK RESET SUBROUTINE

0120   86 08     RESET                 LDA A        #$08        BIT PATTERN

0122   B7 8002                         STA A         $8002      SET PULSE

0125   86 00                           LDA A        #$00        BIT PATTERN

0127   B7 8002                         STA A         $8002      RESET PULSE

012A   39                              RET                      RETURN

                                       END

```

for each step of the analysis cycle. At the end of each step the external clock is reset as is the 8 bit control pattern for the next function. The final step is to activate the AA analysis cycle and to wait for the furnace to cool before injection of the next sample. We have found that programming a waiting time longer than the cooling time extends the life of the graphite cuvettes. The program for operation in the pulsed mode is given in Table 1.

The second injection is a critical part of the analysis cycle for non-metals such as selenium and arsenic. The addition of a co-analyte such as $Ni^{2+}$ has been shown to increase the sensitivity for these elements and reduces loss due to the volatility of these elements [1, 5]. The syringe pump used for the second injection delivers 11.0 ± 0.1 μl/sec of co-analyte. The same syringe pump is used for the total consumption analysis mode. In the total consumption mode, however, the analyte is pumped out of the holding tube into the sampling valve and the dispensing of the sample then proceeds in the same fashion as in the pulsed mode of operation. The software modification for total consumption analysis involves only increasing the second injection time to 4-8 seconds and performing the second injection prior to the sample injection.

The graphite cuvette volume in the currently employed AA system is approximately 50 μl and the standard pulsed mode experiment employs 37 μl ± 1.7 μl analyte from the sampling loop and 11.0 μl + 0.1 μl of co-analyte from the syringe pump. The total consumption mode pumps 100 μl of eluent into the sampling loop and 37 μl are then dispensed into the graphite furnace. This 'overrun' ensures the complete filling of the sample loop and causes no problem in subsequent interpretation of data.

### Conclusion

The use of an inexpensive microprocessor system adds a great deal of versatility to a previously "hard wired" LCAA sampling system [1]. Both pulsed and total consumption analyses are possible with only minor plumbing and software changes. The use of other microprocessor systems would require only a change in machine language. The system has the advantage of being inexpensive. The interface sampling system can be assembled for a component cost of approximately $500 (depending on the availability of surplus equipment).

With the many advantages of performing LCAA analysis on trace level metal-containing compounds, hopefully this technique will find widespread use with investigators in the clinical, environmental, and inorganic biochemistry fields.

ACKNOWLEDGEMENT

We wish to thank April Robertson for technical assistance and the Robert A. Welch Foundation (Grant No. A-694) for financial support.

REFERENCES

[1] Vickrey, T.M. , Buren, M.S., Howell, H.E., *Analytical Letters*, 1978, *A11*, 1075-1095.

[2] Brinckman, F.E., Blair, W.R., Jewett, D.L., and Iverson, W.P., *J. Chrom. Sci.*, 1977, 15, 495-503, and references therein.

[3] Vickrey, T.M., Howell, H.E., Paradise, M.T., *Analytical Chemistry*, submitted.

[4] Slavin, W., Schmidt, G.J., Abstract No. 126, "Pittsburgh Conference on Analytical Chemistry and Applied Spectroscopy", Cleveland, Ohio, March 1979.

[5] Pierce, F.G., and Brown, H.R., *Analytical Chemistry*, 1977, *49*, 1417-1421.

### Assessment of ENI Gemeni microprocessor-controlled centrifugal analyser

**N. Potezny, R.G. White, and T.D. Geary**

*Institute of Medical and Veterinary Science, Box 14, Rundle Street PO, Adelaide, South Australia, 5000.*

The ENI Gemeni was assessed in this institute for its suitability as a general laboratory instrument using a procedure which has been developed during the last two years for the Committee for Evaluation of Kits and Instrumentation of the Australian Association of Clinical Biochemists.

### Materials and Methods

The Gemeni is a miniature centrifugal analyser consisting of an analyser module, microprocessor and work station.

The methods recommended by the manufacturer for use on the Gemeni were run in parallel with routine methods used in this laboratory. These routine methods were:

(1) Glucose – Glucose Analyser, Yellow Springs Inst. Co. (YSI).
(2) Cholesterol – Abbott Agent Enzymatic Reagent, Abbott ABA 100.
(3) Calcium – Cresolphthalein complexone, SMAC, Technicon Equipment Pty. Ltd.
(4) Alkaline
(5) Phosphatase – p-nitrophenol, Beckman TR.

These methods have known accuracies and precisions. The Center of Disease Control, Atlanta, USA, (CDC) hexokinase reference method was used to check the YSI glucose analyser. The cholesterol method has been standardised against the WHO Lipid Standardisation Laboratory at CDC. The calcium method has been compared with the Cali, Young and Bowers [1] method using materials from the Massachusetts Society of Pathology with definitive values assigned by an isotope dilution technique.

The method of assessment was based upon the work of Tonks [2], Barnett and Youden [3], Logan [4], Broughton et al [5] and modified in the light of our own experience.

### Precision

(a) Intrabatch imprecision was checked by the analyses of replicates in the same batch and duplicates on the patient comparisons.

